# Power spectrum, growth velocities and cross-correlations of longitudinal and transverse oscillations of individual *Nicotiana tabacum* pollen tube

**DOI:** 10.1007/s00425-014-2083-5

**Published:** 2014-05-11

**Authors:** Aleksandra Haduch-Sendecka, Mariusz Pietruszka, Paweł Zajdel

**Affiliations:** 1 Laboratory of Plant Physiology, Faculty of Biology and Environment Protection, University of Silesia, ul. Jagiellońska 28, 40032 Katowice, Poland; 2 Institute of Physics, University of Silesia, ul. Uniwersytecka 4, 40007 Katowice, Poland

**Keywords:** Coherence, Energy dissipation, Excitation spectrum, Growth rate, Longitudinal mode, Osmotic potential, Transverse mode

## Abstract

We report on our results concerning growth rate and oscillation modes of the individual pollen tube apex. The observed volumetric growth and growth rate periodicity in the longitudinal (axial) direction are accompanied by transverse oscillations with similar frequencies but higher energies than the axial modes. Examination of the time-domain coherence between oscillations in mutually perpendicular directions revealed minimal energy dissipation in the unperturbed (isotonic) case, opposite to the two remaining cases (hypertonic, hypotonic) with notable correlations. We conjecture that the minimal energy loss is therefore optimal in the natural growth conditions. The longitudinal growth velocity is also found to be the fastest in the unperturbed case. As a result, the isolated system (pollen tube tip) is conserving energy by transforming it from elastic potential energy of extending apical wall to the kinetic energy of periodical motion. The energy dissipation is found to be about 20 % smaller in axial direction than in lateral one, indicating that the main energy consumption is dedicated to the elongation. We further observe that the hypertonic spectrum is shifted towards lower and the hypotonic towards higher frequencies with respect to the isotonic spectrum. In consequence, the turgor pressure inside the growing cell influences monotonically the frequency of both modes of oscillations. The calculated power spectrum seen as a measure of the overall energy efficiency of tip growth under hypertonic, hypotonic and isotonic conditions implies that the biochemistry has been fine tuned to be optimal under normal growth conditions, which is the developmental implication of this work. A simple theoretical extension of the Ortega equation is derived and analysed with respect to its contribution to power spectrum. We show that the plastic term, related to the effective turgor pressure, with maximum contribution at frequency *f* = 0 is responsible for the steady growth. In turn, the elastic contribution dependent on the time derivative of pressure fluctuations tends to move the system into oscillatory mode (*f* > 0). None of those mechanisms is privileged over another. The coupling mechanism is naturally generated through a convolution of those two terms and will decide about the overall character of the growth for each particular case.

**Electronic supplementary material:**

The online version of this article (doi:10.1007/s00425-014-2083-5) contains supplementary material, which is available to authorized users.

## Introduction

The principle of least action (Feynman and Hibbs 1965) is one of the simplest and most beautiful physical cornerstones of biophysical mechanism of cell growth. In its essence, it states that the mechanical system evolves along a path of the least action, which in common sense is considered to be optimal, selecting one mechanical solution over another. In the biological context, a growing cell always follows the optimized growth path under all physiological conditions, similar to the light beam travelling in the shortest time between two points (Fermat’s principle). In this paper, we find on the basis of our measurements and a simple theoretical model that at isotonic conditions the oscillatory growth of an individual male gametophyte of higher plants is additionally optimized by minimizing energy dissipation. We also note that mechanical constraints can self-optimize according to the principle of least action, in this case minimizing energy dissipation.

Pollen tubes are excellent model systems for such study because of their extremely rapid growth rates, non-linear dynamics of oscillatory growth rates (Kroeger and Geitmann 2012a, b), localized exocytosis and growth located in the subapical region (Zonia and Munnik 2008; Zonia 2010). Plant cells are surrounded by a stiff yet elastically flexible wall that is composed of polygalacturonate polymers and cellulose. Cell wall-modifying enzymes such as expansins and pectin methylesterases have demonstrated their role in mediating, respectively, cell wall relaxation, thus promoting growth (Cosgrove 2005; Geisler et al. 2008; Szymanski and Cosgrove 2009). However, expansins have never been shown in vivo to lead to wall loosening and growth.

Water is one of the most important molecules for the emergence and propagation of life. In plants, osmotic pressure is a fundamental property that affects all aspects of the life cycle. Controlled fluid dynamics provides turgor in the plant body. It is utilized to enable work. It drives cell elongation during growth, and it provides the motile force for discharge of male gametes and sperm cells during reproduction (Zonia and Munnik 2007).

Currently accepted models for plant cell growth consider two key factors: cell wall relaxation and turgor pressure being responsible for cell elongation. However, the controversy remains, which of them acts as principal stimulus. One approach considers cell wall relaxation being controlled independently of turgor pressure and acting as a prerequisite to turgor-driven cell expansion. The molecular details of cell wall growth have been characterized recently in extensive work on the green algae *Chara* (Proseus and Boyer 2007, 2008; Boyer 2009). In the second approach, the turgor pressure induces tension in the wall, leading to the elevated strain in the Ca^2+^ pectate bonds, which then lose calcium to newly secreted pectins that are appressed to the wall by osmotic pressure. The decreased density of calcium pectate bonds in the wall leads to cell wall loosening, enabling pressure-driven elongation and intercalation of the newly secreted calcium pectate polymers into the growing wall. Some studies show that when pressure drops below a critical value, the growth ceases altogether (Boyer 2009). The cell wall of the subapical growth zone and the apical dome are composed primarily of pectin polymers (Bosch and Hepler 2005; Parre and Geitmann 2005; Zonia 2010). Experimental manipulation of the extracellular osmotic potential induces rapid water flux across the plasma membrane into (hypotonic treatment) or out of (hypertonic treatment) pollen tubes (Zonia and Munnik 2004; Zonia 2010). Hydrodynamics affects pollen tube growth rates, growth rate oscillation frequencies and amplitudes (Kroeger et al. 2011, Fig. 4), and the rates of exocytosis and endocytosis (Zonia et al. 2006; Zonia and Munnik 2008; Zonia 2010). These results may suggest that oscillations in osmotic pressure would result in oscillations in exocytosis, leading to oscillations in secretion, wall loosening, intercalation of new pectin polymers into the cell wall, ultimately resulting in pollen tube growth oscillations. Recent work presented the first evidence that turgor pressure oscillates (‘hydrodynamic model’, as proposed by Zonia) during growth of lily pollen tubes (Zonia and Munnik 2011). Nonetheless, the opposite view about the primary role of wall strain/stress relations and periodical wall assembly is more commonly accepted (Winship et al. 2010, 2011). In our opinion, this ongoing controversy is somewhat artificial, since only a full self-consistent model, which includes non-trivial coupling between both subsystems, might deliver a proper solution.

In this work, we would like to concentrate on the less explored areas, namely the dissipation and character of the oscillatory growth, as revealed by the power spectrum. Power spectral density (PSD), or power spectrum for short, describes how the power of a signal or time series is distributed over the different frequencies. Here, power can be the actual physical power, or more often, for convenience with abstract signals, can be defined as the squared value of the signal.

The main experimental concern is the non-linear pressure/frequency dispersion relation and new features encountered in the excitation spectrum of pollen tubes and their variations under hyper- and hypo-tonic conditions. We also introduce a simple two-parameter model with constant turgor pressure and fluctuating osmotic pressure, to recognize the control of non-linear growth behaviour.

## Materials and methods

### Pollen culture and extracellular osmotic stress treatments

*Nicotiana tabacum* pollen was used for these studies (Zonia and Munnik 2004; Zonia et al. 2006). Anthers were harvested immediately before dehiscence and placed in desiccation chambers between 18 and 24 h. Tobacco pollen was collected and stored at −20 °C. Pollen from seven plants was harvested in batches and analysed before experiments for germination, morphology and growth rates. We chose two very similar pollen samples and used them for all the studies to ensure uniformity of experimental material. After removal from −20 °C, cells were held at room temperature for 25–30 min. before suspending in standard germination medium [6 % (w/v) sucrose, 1.6 mM H_3_BO_3_, 200 μM CaCl_2_, and 25 μM MES (pH 5.5)]. Pollen was germinated and grown in culture chambers where it was assembled on microscope slides using silicone isolators for 3 h at 22.5 °C before performing experiments on slabs containing 0.3 % (w/v) low gelling temperature agarose (plant cell culture grade, Type VII, Sigma) in 200 μl germination medium. Pollen was used for imaging from 3 to 5 h after start of germination. Imaging was performed on a Zeiss Axiophot upright microscope, connected to a Nikon DXM/200 or UltraPix CCD camera, which were run by NSI-Elements or UltraView morphometric software, respectively, to record information about the experiments (Centre for Advanced Microscopy, Amsterdam, The Netherlands).

**Fig. 1:**
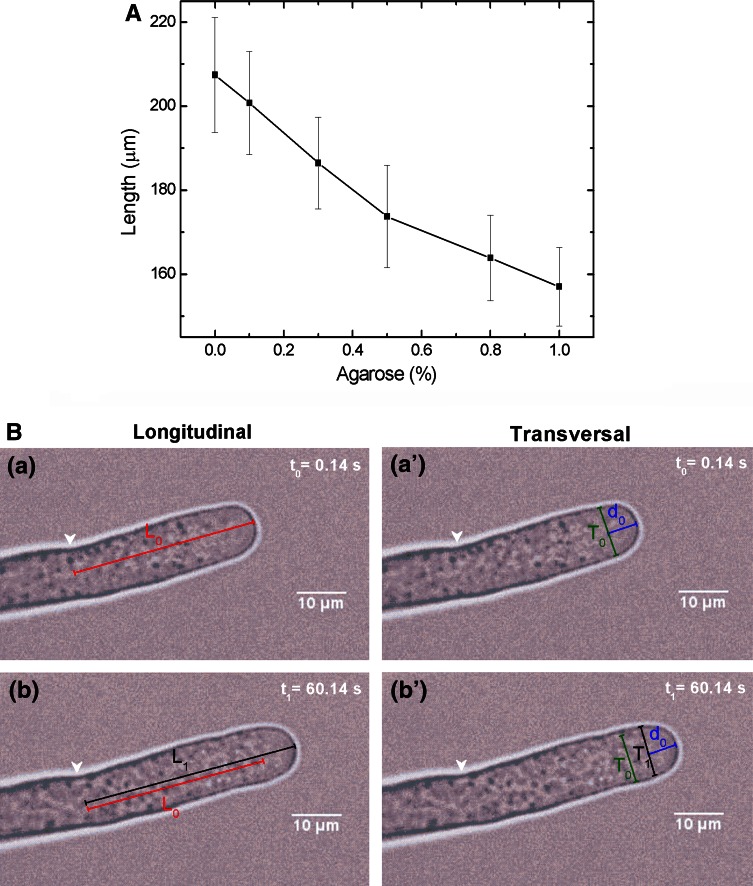
**a** Average of ten samples’ (pollen tubes) lengths for different concentrations of agarose in tobacco germination medium. *Error bars* correspond to 2*σ*. **b** Fixed reference system measurement method (*a*, *b*) versus co-moving reference system (*a*′, *b*′) of the same pollen tube apical region. *L*_0(1)_ and *T*_0(1)_ stand for longitudinal and transversal amplitudes; *d*_0_ = 6 μm is a constant assumed radius at which *T* is read off in the co-moving frame. *Scale bar* and the *arrow* pointing to the origin of the fixed reference system are indicated. Calculated spatial resolution ratio equals 0.226 ≈ 0.23 μm/px

The main goal was to analyse the response (in real time) of single pollen tube to changes in the extracellular osmotic potential. To do these experiments, an appropriate technique was developed to immobilize the cells, so they would not move during the experiment. The method to grow the cells in an agarose gel medium directly on microscope slides was used. Firstly, growth of test populations of pollen tubes using different concentrations of agarose was measured and recorded. The results showed that a concentration of 0.3 % agarose (see Supplementary Information) gave the best growth but still kept the cells immobile (Fig. 1a). Next we tested, how long it would take for the osmotic treatments to penetrate through the agarose medium. It was found that the osmotic treatments diffused through the agarose medium within 12–15 s. That was rapid enough for the live cell studies. The experimental method involved recording fast time series images of the live pollen tubes before and after osmotic treatment. Microscope images were collected and processed at a rate of 1 image per 3 s. The recordings started 5 min before the specific osmotic treatment were added and refocused on the same cell (this took from 30 s to 1.5 min). Then the pollen tube growth response was recorded for 10 min. The images were then saved and transferred to a computer for further analysis. Experiments with environment conditions close to isotonic conditions (hereafter called isotonic) were induced by addition of 100-μl standard germination medium without agarose on top of microscope slides with pollen gel culture. For the other specific osmotic treatment, liquid germination medium was removed from inside of the silicon isolator and 100 μl of appropriate substance was added. Experiments with hypo-osmotic stress (here we follow the nomenclature used in Zonia and Munnik 2011), were induced by the addition of water to the gel cultures, so that a 100 % (v/v) water stress treatment is a 1:2 (v/v) dilution of the pollen tube culture. Experiments with hyper-osmotic stress were induced by addition of 25 mM NaCl. Although NaCl is biologically active and is metabolized in a cell, what potentially can lead to the change of the pressures, such environment was found earlier to give stationary conditions over the time span (~5 min) of the experiment (compare Zonia and Munnik 2008).

The growth rate of the cells was measured using the open-source morphometry software ImageJ in 3-s intervals independently from the used camera. Longitudinal (*L*) growth (mainly depending on plastic deformation of the wall) was measured by choosing a fixed point on the cell, and then finding the distance to the apex (fixed reference system, Fig. 1b). Transversal (*T*) size change (principally elastic as the transversal size does not increase significantly) was measured at a constant distance from the apex (reference system co-moving with the tip, Fig. 1b) because this apical region of the pollen tube undergoes the most rapid and extensive cell oscillations during osmotic perturbation experiments. These measurements gave a list of lengths (micrometre) that were imported into the SigmaPlot software. The exact time intervals were received from UltraView/NSI, which were imported into the same worksheet in SigmaPlot. From length and time measurements, growth rate was calculated using the formula (*l*_1_ − *l*_0_)/(*t*_1_ − *t*_0_), with *l* for length (width) and *t* for time in both mutually perpendicular (longitudinal and transversal) cases, respectively. Growth rates obtained in that procedure were later on used to establish phase relations between longitudinal and transversal oscillation modes.

We have to note that the observed changes in the transversal mode are on the edge of the detection and optical resolution of the system. However, there are two factors that allow us to justify the relevance of the data:

- the analysis of correlations between the *L* and *T* modes displays correlation, which could not originate only from the thermal or optical noise of the system, as it is improbable to generate a correlated noise in two different places for such extended time period,
- there are significant regularities observed in power spectrum of both modes.

For further detailed description of the employed measurement routine—see Supplementary Information.

### Power spectra evaluation

PSD, the fast Fourier transform (FFT) and correlation functions of longitudinal and transverse oscillation modes were obtained using algorithms built into the Origin (Microcal) plotting software. The Fourier transform relates the function’s time domain to the function’s frequency domain. The component frequencies, spread across the frequency spectrum, are represented as peaks in the frequency domain. The oscillation data were not filtered before analysis to preserve all the information including noise, ubiquitous in biological systems. Most computations and plots were performed using Excel (Microsoft), SigmaPlot 8.0 (SPSS Science Inc., Chicago, IL, USA) and Origin (Microcal). In case of longitudinal oscillations the bias (identified as the mean growth velocity) was subtracted before FFT calculations. To reduce termination artefacts, the Hann window function was used.

PSD was calculated for three different osmotic environments and two mutually perpendicular vibration modes (longitudinal and transversal), and the corresponding total energy was calculated. The most intense peaks were indicated and the appropriate frequencies assigned. (On the use of Fourier transformation to analyse pollen tube growth oscillations, see also Sanati Nezhad et al. 2013).

The Nyquist sampling theorem was satisfied in the time domain. The signal with total length of 300 s can sufficiently be described in the frequency domain with low frequency cut-off at 2 * 1/300/s ≈ 0.007 Hz. That means that the mode with the longest period is fully contained at least twice in our measurement. To be on a safe side we did not consider any signals below 0.013 Hz, which is an equivalent of four complete periods of oscillation. We have to note that the 0.013 Hz lies very close to the main period of oscillations reported earlier (Zonia et al. 2006). Later on, we shall refer to it by calling it Ω_0_.

On the other hand, the signal sampled every 3 s (sampling rate 0.33 Hz) can sufficiently be described in the frequency domain with the Nyquist frequency of 0.17 Hz = 1/(2 * 3 s). We have to note that at least six clearly identified modes of oscillations were contained within the range (0.015, 0.09) Hz, i.e. with at least quadruple oversampling.

A linear regression was applied to all identified modes in the frequency spectrum. In all cases, the determination coefficient *R* ^2^ exceeded 0.99 confirming high correlation of all oscillation modes. We have to note that it does not exclude any lower frequencies present but they are outside the safe detection window. Basic oscillation periods were calculated in all cases from the simple formula *T*_base_ = 1*/f*_base_ and expressed in seconds. To get an idea about the possible shape of the signals in the time domain, the identified modes were later on used for the inverse Fourier transform with no assumed phase shift (*φ*_i_ = 0):

$$f(x) = \frac{{A_{0} }}{2} + \sum\limits_{i = 1}^{N} {A_{i} } \sin (2\pi f_{i} t + \phi_{i} ).$$

The time cross-correlation function, defined as

$${A\left( \tau \right)} = \int\limits_{0}^{\tau } {L\left( t \right)T\left( {t + \tau } \right)} {\text{d}}t$$

where *L* and *T* denote longitudinal and transversal oscillation modes, respectively, was calculated for the 9-point adjacent average of the input data. In all cases the data were detrended. We have to note that forgetting to remove the bias may introduce unwanted artefacts in the frequency domain (like imitation of 1/*f* noise from the *f* = 0 peak) or a broad correlation peak in the time domain. Similar procedure was performed for isotonic and perturbed treatments to find the relative time shift between the longitudinal and transversal modes.

### Model of the energy spectra of pollen tube dynamics

A model of pollen tube growth must include terms for turgor pressure, elastic and plastic deformation of the cell wall, and incorporation of new materials into the wall. The goal of the present analysis is to derive a model that relates changes in osmotic pressure to growth dynamics. The growth of cell walls is determined by the visco-elastic and plastic response of the wall to the mechanical force exerted by the effective turgor pressure. To decipher behaviour specific for the processes involved we begin with the Ortega (1985) equation

1 $$\frac{1}{V}\frac{{{\text{d}}V\left( t \right)}}{{{\text{d}}t}} = \varPhi \left( {P\left( t \right) - Y} \right) + \frac{1}{\varepsilon }\frac{{{\text{d}}P\left( t \right)}}{{{\text{d}}t}}$$

which formalizes the combined effect of plastic (*Φ*) and elastic (*ε*) ingredients on the cell volume *V*. It takes into account elastic deformation of the cell wall by introducing volumetric elastic modulus *ε* for the changing turgor pressure *P*(*t*) with the constant yield threshold *Y*; *Φ* stands for the ‘extensibility coefficient’ of the wall which is defined as the inverse of the viscosity of the material. Equation (1) describes the relative rate of change in volume of cell wall chamber, as the sum of irreversible and reversible deformation of the wall.

To begin, we consider the volume of the growing pollen tube and utilize the property of additivity to partition the total cell volume *V*_T_ into two compartments *V*_D_ and *V*_A_. *V*_D_ is the volume of the pollen tube distal region; *V*_A_ is the volume of the apical region (Zonia et al. 2006), and *V*_T_(*t*) = *V*_D_(*t*) + *V*_A_(*t*) is a function of time *t*. Experimental data indicate that there is a continuous smooth transition in the cell mechanical properties between the apical and distal regions (Geitmann and Parre 2004; also Pietruszka et al. 2012, Fig. 2A, B; Vogler et al. 2013). We further assume that *V*_D_(*t*) = *u*_0_ *t* and [*u*_0_] = μm^3^/s, where *V*_D_ is considered for the quasi-linear phase of elongation growth (e.g. Pietruszka 2012, Fig. 8A) where this approximation holds. Next, to describe pollen tube apical volume oscillations we utilize Eq. (1) which is a first-order differential equation that takes into account elastic (*ε*) deformation of the cell wall. Further, we expand this equation by allowing time dependence of the extensibility coefficient *Φ* = *Φ*(*t*) while keeping the elastic modulus *ε* constant (it may be treated as a piecewise constant function along the tube) in the apex. We assume that the intracellular osmotic pressure can change in response to changes in the extracellular osmotic potential, therefore the osmotic pressure term *δπ* = *δπ*(*t*) must be also introduced in Eq. (1). We also make a distinction between the turgor pressure *P* which is maintained constant and time-dependent osmotic pressure fluctuations *δπ*(*t*) = ±[*π*_out_(*t*) − *π*_in_(*t*)], which are variable inside or outside the vacuole. As a result, we construct a so termed “two-fluid model”: *P* ± *δπ*(*t*). Assuming turgor pressure *P* = constant and turgor threshold *Y* = constant, we may rearrange Eq. (1) in the following way

2 $${\frac{1}{{V_{\text{A}} }}}\frac{{{\text{d}}V_{\text{A}} \left( t \right)}}{{{\text{d}}t}} = \varPhi \left( t \right)\left( {P - \delta \pi \left( t \right) - Y} \right) - \frac{1}{\varepsilon }\frac{{{\text{d}}\left( {\delta \pi \left( t \right)} \right)}}{{{\text{d}}t}}$$

since both *P* and *Y* time derivatives vanish. We have to note that constant character of turgor pressure as suggested by Benkert et al. (1997) is not strictly what has been shown later by Zonia and Munnik (2011) (see Fig. 3 ibid.); however, its time derivative is negligible.

Equation (2) shows that the relative change in time of the apical volume *V*_A_ depends on the sum of the plastic properties proportional to the effective pressure *P* – *δπ* − *Y*, and the elastic properties related to the changes (the time derivative) of the osmotic pressure. It is important to observe, that identical formalism may be restricted to the formulae including only the fluctuations of turgor *δP*(*t*), without the osmotic pressure *δπ*(*t*).

From the formal point of view, the whole calculus above, repeated after such substitution, leads to the same kind of equations. The material constants responsible for cell wall properties of the growing wall are specified by *Φ* and *ε*, describing irreversible growth and reversible cell extension, respectively. Furthermore, by taking *Φ* = *Φ*(*t*) and *ε* = constant, we may follow (Pietruszka 2013) to introduce some additional cell wall properties. Cell wall visco-elasticity is expressed with the relation *Φ*(*t*) = *Φ*_0_ *x*_0_exp(−*k*_0_ *t*), where *Φ*_0_ = constant is the extensibility coefficient as originally proposed by Lockhart (1965), with dimensionality [10^−6^/(MPa s)]. The quantities *x*_0_ and *k*_0_ [1/s] represent polymer initial (potential) density *x*_0_ = *x*(*t* = *t*_0_) and cell wall intercalation rate, respectively. By substituting the explicit form of *Φ*(*t*) in Eq. (2) we get

3 $${\frac{1}{{V_{\text{A}} }}}\frac{{{\text{d}}V_{\text{A}} \left( t \right)}}{{{\text{d}}t}} = \varPhi_{0} x_{0} \left( {P - \delta \pi \left( t \right) - Y} \right){\text{e}}^{{ - k_{0} t}} - \frac{1}{\varepsilon }\frac{{{\text{d}}\left( {\delta \pi \left( t \right)} \right)}}{{{\text{d}}t}}.$$

Equation (3) represents a differential equation that takes into account the existing pressures in the system and also (in the first approximation) the cell wall elastic and plastic properties. This equation introduces deposition of new wall material into the existing cell wall at a rate *k*_0_, with the initial concentration of pectins that are used for new cell wall growth denoted as *x*_0_. We recall that the pollen tube growth is described by its total volume *V*_T_ (*t*) [growth rate is then defined by the time derivative: *V′*_T_ (*t*) = *V′*_D_(*t*) + *V′*_A_ (*t*)] and that the continuity condition for the volume flow from the apical to distal region must be fulfilled. The general solution of such an equation is non-trivial but we shall only concentrate on the Eq. (3) as it describes the relative growth rate of the apex of pollen tube.

Now we offer a qualitative evaluation of the Fourier decomposition of Eq. (3) and its implications to biological processes. The right-hand side of Eq. (3) can be split into three contributions: plastic, fluctuating-plastic and fluctuating-elastic, respectively

4.1 $${\varPhi_{0} x_{0} }\left( {P - Y} \right){\text{e}}^{{ - k_{0} t}} \sim {\text{e}}^{{ - k_{0} t}}$$

4.2 $${\varPhi_{0} }x_{0} \delta \pi \left( t \right){\text{e}}^{{ - k_{0} t}} \sim \delta \pi \left( t \right){\text{e}}^{{ - k_{0} t}}$$

4.3 $${\frac{1}{\varepsilon }}\frac{{{\text{d}}\left( {\delta \pi \left( t \right)} \right)}}{{{\text{d}}t}}\sim \frac{{{\text{d}}\left( {\delta \pi \left( t \right)} \right)}}{{{\text{d}}t}}.$$

Their respective Fourier transforms will have forms (*f* stands for frequency):

5.1 $${\frac{1}{{k_{0} + 2\pi if}}}$$

5.2 $${F\left( {\delta \pi \left( f \right)} \right)}*\frac{1}{{k_{0} + 2\pi if}}$$

5.3 $${ifF\left( {\delta \pi \left( f \right)} \right)}$$

where ‘*’ symbolizes convolution, and *F* Fourier transform.

The contributions to the energy distribution spectrum read:

6.1 $${\frac{1}{{k_{0}^{2} + 4\pi^{2} f^{2} }}}$$

6.2 $${\left| {F\left( {\delta \pi \left( f \right)} \right) * \frac{1}{{k_{0} + 2\pi if}}} \right|}^{2}$$

6.3 $${f^{2} }\left| {F\left( {\delta \pi \left( f \right)} \right)} \right|^{2} .$$

Let us briefly analyse these components. The first one (more viscous, plastic) will have a maximum at (about) *f* = 0 (quasi-linear) and a Lorentz form for higher frequencies (compare with Kroeger and Geitmann 2013). It is very unlikely to contribute to oscillations and the central part will be subtracted by detrending before Fourier procedure. It is worth noting that any appearance of oscillations (*f* > 0), will compete for a spectral density allocated to this part. To discuss the remaining contributions let us simplify for a moment the case by assuming that *δπ*(*t*) is a random-type noise with 0 (detrended) expected value. In biological systems, its power spectrum will usually have a form *F*(*δπ*) = *S*_0_/*f* ^−α^, where *S*_0_ is a power per frequency unit and 1 < *α* < 2 (*α* = 2 for Brownian or *α* = 1 for “pink” noise) (see Dutta and Horn 1981; Weissman 1988, for a review). The second component (fluctuating-plastic) is a non-trivial one and will not be discussed here in detail but will act as an effective coupling between the first and last terms.

On the other hand, the third term (fluctuating-elastic in origin) will have an envelope of *f* ^2^ *F*(*δπ*), which for the noise spectrum assumed above will read *f* ^*β*^ *S*_0_, where 0 < *β* < 1. This will necessary be 0 at *f* = 0 (even for *β* = 0, the noise must have a lower energy cut-off) and increase with frequency, therefore, it will naturally promote the oscillatory state. We have to note that in the current form, none of those terms selects a clear “resonant” value for frequency. It will most likely be decided by the coupling term or an external constraint.

## Experimental results

Longitudinal (*L*) and transverse (*T*) oscillations of the apical region of *N. tabacum* pollen tube were measured under hypertonic (25 mM NaCl), isotonic and hypotonic (H_2_O) conditions. Careful measurements revealed oscillations not only in the axial direction, but also in the lateral one. Instead of the usual applied co-moving frame reference system, a fixed coordinate system was used (Fig. 1b) to reveal phase relations between mutually perpendicular spatial modes of oscillations and retrieve various key characteristics of the periodic pollen tube growth.

**Fig. 2:**
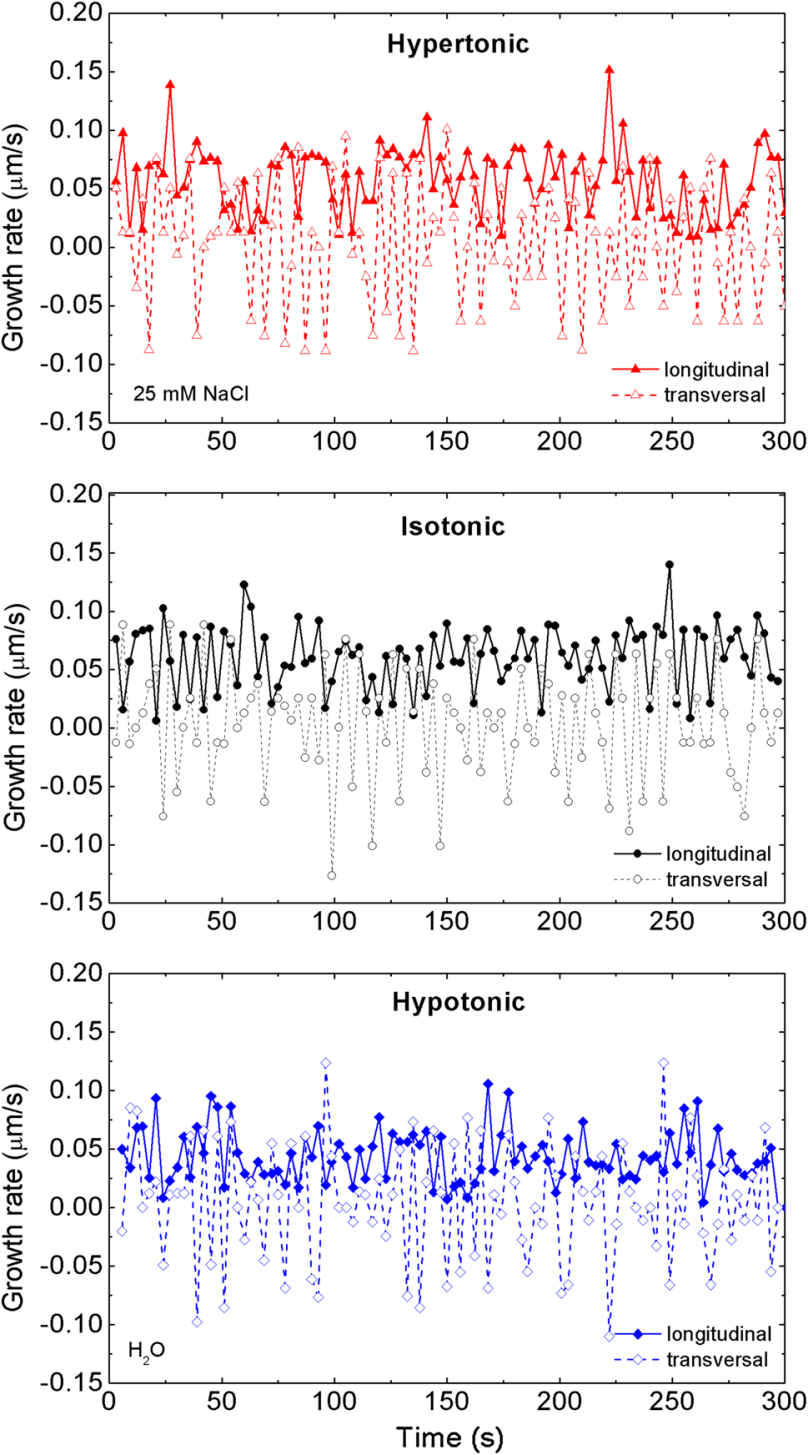
Growth rate oscillations of *Nicotiana tabacum* single pollen tube plotted from the raw experimental data (without averaging). Two oscillation modes, longitudinal and transversal, are presented for hypertonic, isotonic and hypotonic treatment. The calculation of growth rate is acquired with the optimal temporal resolution (sampling) of 3 s. Here we have 0.45 μm/3 s corresponding to 0.15 μm/s in the plot

Raw measurements are plotted in Fig. 2, for *L* and *T* oscillation modes under all growing conditions. We have not applied any averaging before the spectral analysis as it effectively acts as a high frequency filter. At the first sight, it is easily seen that the transverse oscillations possess, on average, slightly greater amplitudes than the longitudinal ones. The tendency is clearly visible in the values collected in Table 1. The amplitudes of *L* oscillations decrease with decreasing pressure but behave non-monotonically for *T* with the smallest amplitude in the isotonic case. Further spectral analysis using the FFT, required subtraction of bias (baseline) in all three longitudinal cases. By identifying bias with mean (linear) elongation velocity, we found it to be the fastest for isotonic case (Table 2) based on the calculations with the 95 % confidence level.

**Table 1:** Calculated oscillation amplitudes for hypertonic, isotonic and hypotonic cases

Amplitude (μm/s)	Hypertonic	Isotonic	Hypotonic
Longitudinal	0.0289	0.0278	0.0222
Transversal	0.0514	0.0477	0.0506

**Table 2:** Calculated mean velocity (growth rate in μm/s) and the confidence interval (left-, right-edges) for longitudinal oscillation modes in hypertonic, isotonic and hypotonic cases

Growth rates	*N*	Mean (μm/s)	Left edge	Right edge
Hypertonic	15	0.045 (7)	0.040	0.049
Isotonic	15	0.058 (10)	0.052	0.065
Hypotonic	15	0.033 (18)	0.023	0.044

Confidence level: 1 − α = 0.95 (95 %), error: 2σ

We have to stress that *L* and *T* oscillations cannot be treated as the same mode. Their frequency and amplitude characteristics clearly show that they must be considered as separate degrees of freedom, although highly coupled (Fayant et al. 2010, Fig. 1).

Spectral analysis also delivered reliable data concerning energy consumption/dissipation by the growing system. Performing integration over power spectra, we calculated total energy of oscillations, and hence contributions of longitudinal and transverse modes (Table 3). The calculated total energy in the isotonic case was the lowest of all cases, fulfilling the fundamental physical principle of the least action for the pollen tube growing in the natural conditions. Moreover, the computed transversal oscillations were of about one order of magnitude more powerful than the longitudinal ones. It meant that the energy connected with longitudinal motion is less dissipated in oscillations and mostly directed to the elongation.

**Table 3:** Calculated total energy for longitudinal and transversal oscillations modes in hypertonic, isotonic and hypotonic cases

Energy (μJ)	Hypertonic	Isotonic	Hypotonic
Longitudinal	(5.3 ± 0.5) × 10^−8^	(4.5 ± 0.4) × 10^−8^	(3.1 ± 0.3) × 10^−8^
Transversal	(1.7 ± 0.2) × 10^−7^	(1.4 ± 0.1) × 10^−7^	(1.6 ± 0.2) × 10^−7^
Total	(2.2 ± 0.2) × 10^−7^	(1.9 ± 0.2) × 10^−7^	(1.9 ± 0.2) × 10^−7^
Longitudinal (%)	24.0	23.7	16.1
Transversal (%)	76.0	76.3	83.9

Energy dissipation (in %) for the longitudinal and transversal modes indicated

**Fig. 3:**
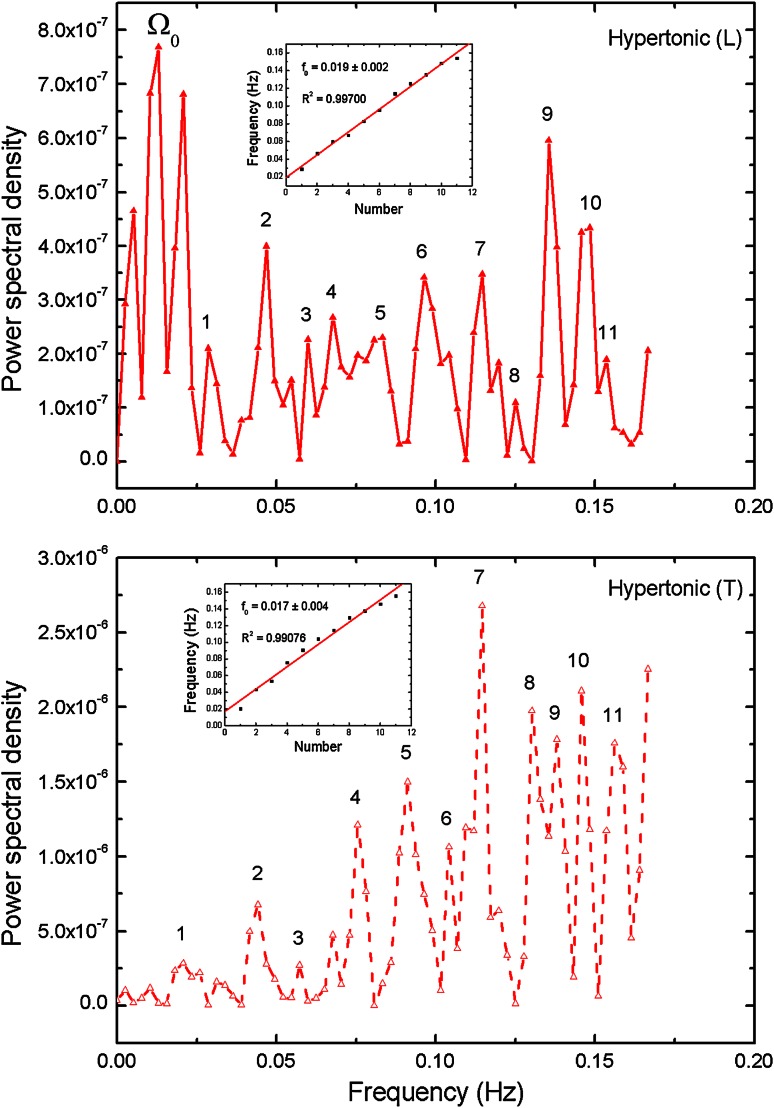
Power spectral density (PSD) calculated for longitudinal (*L*) and transversal (*T*) oscillation modes for the hypertonic data presented in Fig. 2. The strongest peaks are indicated and the corresponding frequencies assigned. The *inset* presents linear dependence of higher harmonics and linear regression fit, which slope is equivalent to the basic frequency (*R* ^2^ delivers determination coefficient; *P* < 0.0001 in all *insets* in Figs. 3, 4 and 5). Calculated total energy: see Table 3

**Fig. 4:**
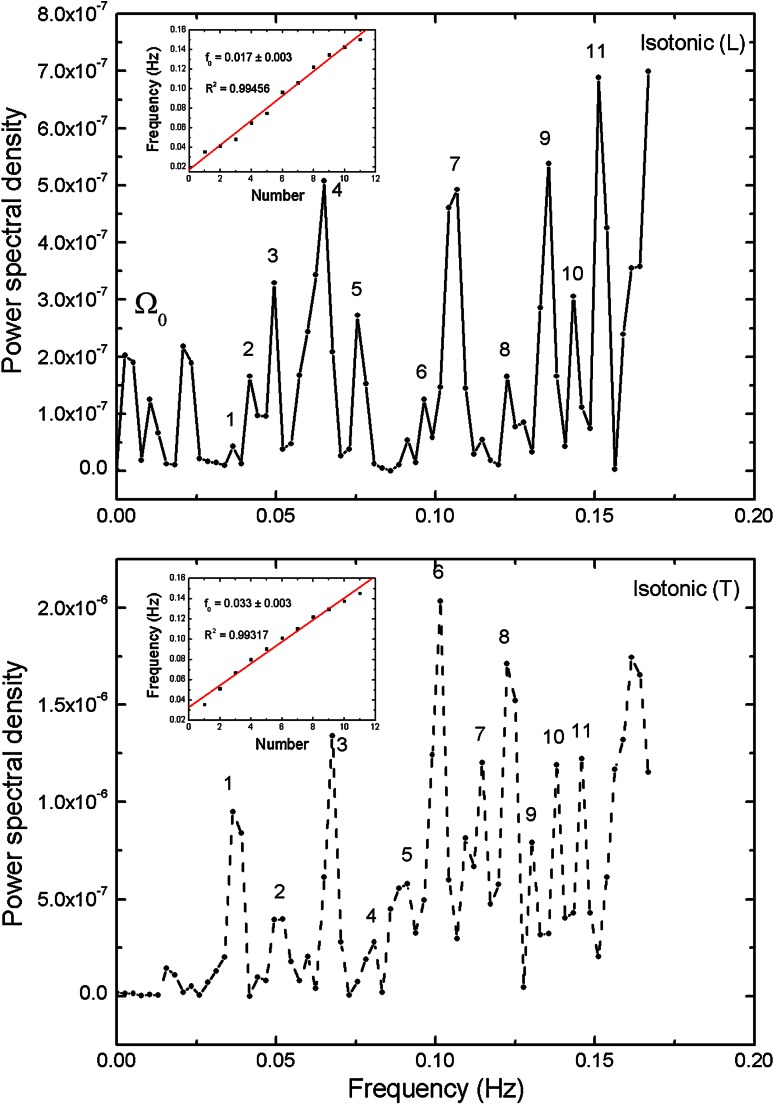
Power spectral density (PSD) calculated for longitudinal (*L*) and transversal (*T*) oscillation modes for the isotonic data presented in Fig. 2. Further description as in Fig. 3

**Fig. 5:**
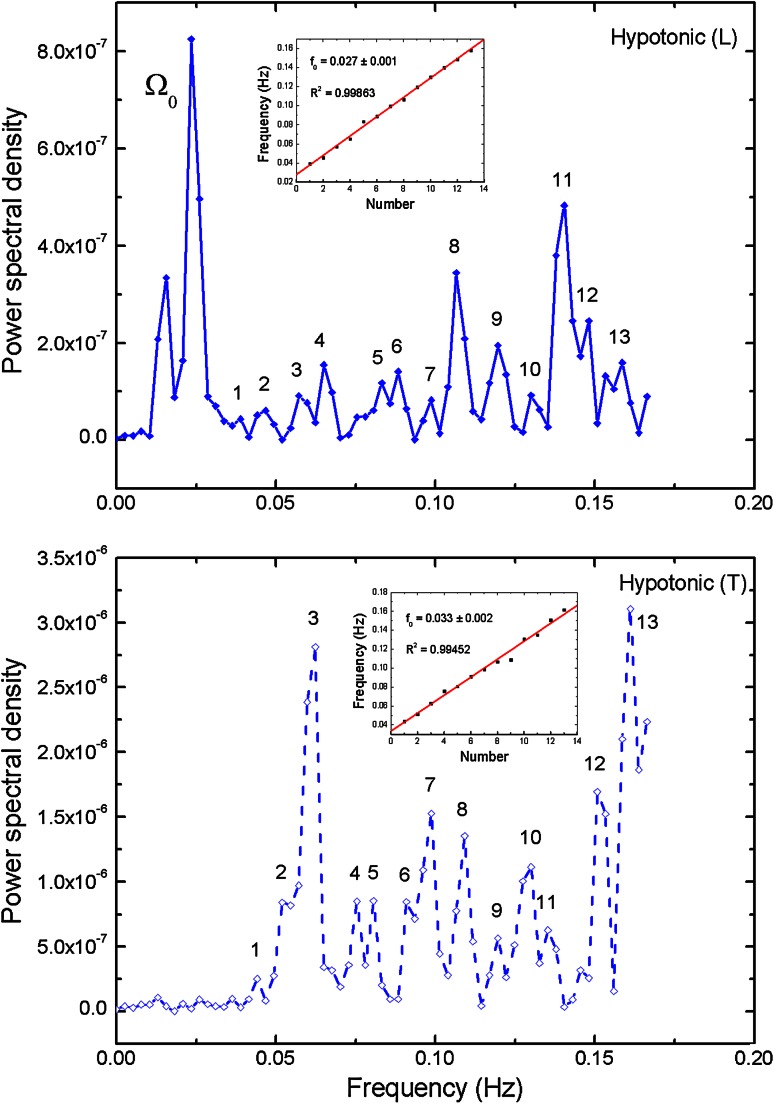
Power spectral density (PSD) calculated for longitudinal (*L*) and transversal (*T*) oscillation modes for the hypotonic data presented in Fig. 2. Further description as in Fig. 3

The Figs. 3, 4 and 5 present power density spectra calculated for *L* and *T* oscillation modes for the original hypertonic, isotonic and hypotonic data presented in Fig. 2. The insets visualize positions of the noticeable maxima frequencies together with linear fit *f* = *f*_base_ +*nf*_*0*_. We have to note that the base frequency might suffer from the possible omission or low amplitude of the lowest frequency peaks, which would change the numbering of the harmonics. In turn, the *f*_0_ obtained from the slope of the linear regression fit did not depend on the choice of the first peak. The values are presented in Table 4 (*T*_base_ = 1/*f*_base_). The calculated determination coefficients *R* ^2^ are equal or above 0.99 confirming linearity of frequencies. The shapes of the power spectra were found to be different for the *L* and *T* cases in the low-lying pronounced peaks present in the *L* case, which were absent in the *T* case. They have been marked as Ω_0_ and can be related to the peaks in spectrum observed by Zonia et al. (2006). A wide gap near zero frequency was found in all of transversal cases, while there were low frequency peaks present in longitudinal cases with the smallest one observed for isotonic one. In any case, all peaks above 0.015 Hz for transversal mode possess their counterparts in longitudinal case—the mutually corresponding frequencies being almost equal. All frequency spectra have no intensity close to zero frequency, resulting from baseline subtraction.

**Table 4:** Calculated basic frequency modes and corresponding oscillation periods

Periodicity	Hypertonic		Isotonic		Hypotonic	
	*f*_base_ (Hz)	*T*_base_ (s)	*f*_base_ (Hz)	*T*_base_ (s)	*f*_base_ (Hz)	*T*_base_ (s)
Longitudinal	0.019 (2)	53 (6)	0.017 (3)	59 (10)	0.027 (1)	37 (2)
Transversal	0.017 (4)	59 (14)	0.033 (3)	30 (3)	0.033 (2)	30 (2)

In addition, under hypotonic conditions, when the pressure is greatest, the frequency is also the highest. This fact can be easily explained by the total turgor pressure value, which is the greatest in this case.

**Fig. 6:**
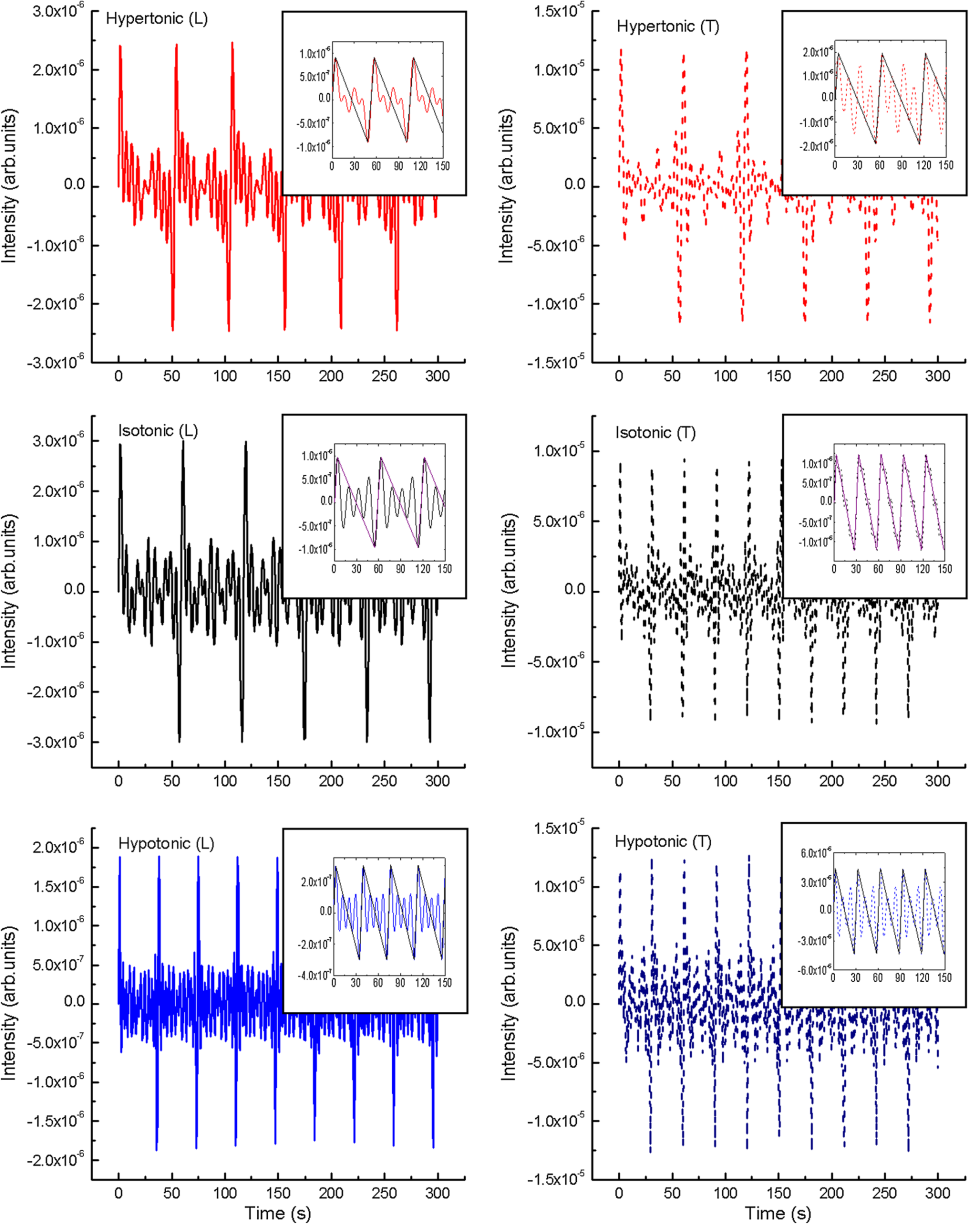
Calculated inverse Fourier transform. Data presented for the sum of the first 11 harmonics [hypertonic (*L, T*), isotonic (*L, T*)] and 13 harmonics [hypotonic (*L, T*)]. The sum of four first harmonics (*insets*) calculated for data indicated in Figs. 3, 4 and 5. The *underlying* *triangle* waveform, corresponding to wall building/relaxation processes, indicated

Figure 6 depicts the results of the inverse Fourier transform computations of selected modes. In Fig. 6 we have taken the simple sum of the first 11 harmonics for hypertonic and isotonic cases and 13 harmonics for hypotonic one, to uncover periodicity hidden in Fig. 2. These results reveal the main period of all considered modes of oscillations in the time domain. In the following example (Fig. 6, the insets), we considered the sum of 4 first harmonics to retrieve the character of the basic signal. It turned out that it could approximately be described by triangular jigsaw: from steep increase in the hypertonic case to the moderate increase in the hypotonic case.

**Fig. 7:**
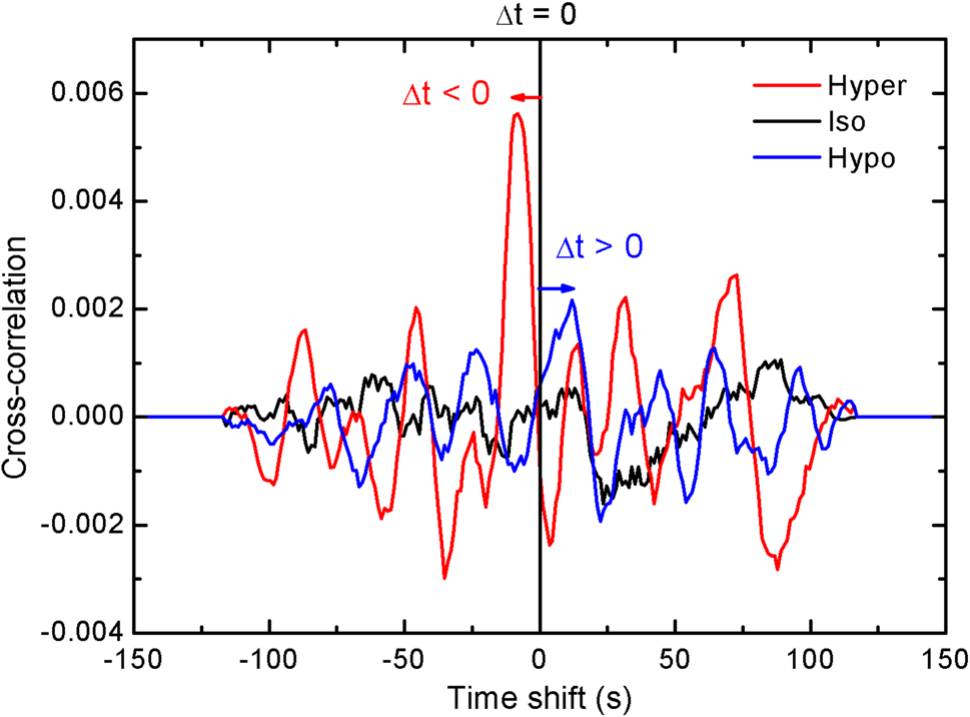
Cross**-**correlation function for longitudinal (*L*) and transversal (*T*) oscillation modes presented for hypertonic, isotonic and hypotonic treatment. The correlation was calculated for a 9-point adjacent average of the time signals. Δ*t* is a time shift between *L* and *T* modes

We also examined the *LT*-correlation functions under hypertonic, isotonic and hypotonic conditions (origin). For this case, the signals were mapped from −150 to +150 s, over a total range of 300 s, Fig. 7. The detailed analysis of such correlation cross-product is beyond the scope of this paper but we have to observe that the amplitudes in the perturbed cases seem to be much higher than in the unperturbed one.

## Discussion

In this study, we will focus on mechanical aspects of periodical growth of *N. tabacum* pollen tube. The essential feature of the study was to investigate general energetics of the less studied transverse mode (the transverse component is not commonly measured, but recent studies used a parameterization of the cell surface (e.g. Rojas et al. 2011), and the correlations between *T* and *L* oscillations of the apical part of the cell. We do not wish to argue with the biochemical or intracellular mechanisms of the cell growth preferring turgor pressure or cell wall relaxation, as they are present on the equal terms in the calculations conducted in the theoretical section.

From our point of view there are several main areas of interest: amplitudes, frequencies, energies and cross-correlations, and their influence on the growth rate. First of all, the linear growth velocity (corresponding to the linear extension of the distal volume *V*_D_) is the fastest for the unperturbed (isotonic) case. This hypothesis: *v*_hiper_ < *v*_hypo_ < *v*_iso_, with *v* standing for the linear mean velocity is found to be true at the 95 % confidence level, on the basis of data from Table 2. This linear velocity can be treated as an indirect measure for the rate of wall building processes and exocytosis at a given turgor pressure *P*, providing that the elastic component can be neglected. Clearly the steady growth component reflects the *f* = 0 power spectrum contributed by plastic deformation.

We must note that the optimal character of the isotonic solution cannot be justified only on the basis of the longitudinal oscillatory mode. Table 1 shows that that the amplitudes of *L*-mode oscillations change monotonically with pressure as well as their energies (Table 3). Hence, it is not clear why the isotonic case is optimal, especially when iso- and hyper-tonic cases have also very similar values (Tables 4, 5). The situation changes when we take into consideration also the transverse mode, which is clearly singled out by amplitude in Table 1 and a little weaker by energy in Table 3. Furthermore, the base frequencies of *T* oscillations are now closer to hypo-osmotic case.

**Table 5:** Calculated relative elongation (in %) per cycle in basic osmotic environments; *l*_0_ = *l*(*t* = 0), *l*_1_ = *l*(*t* = *T*)

Periodicity	Hypertonic		Isotonic		Hypotonic	
	*f*_base_ (Hz)	*T*_base_ (s)	*f*_base_ (Hz)	*T*_base_ (s)	*f*_base_ (Hz)	*T*_base_ (s)
Longitudinal	0.019 (2)	53 (6)	0.017 (3)	59 (10)	0.027 (1)	37 (1)
(*l*_1_ − *l*_0_)/*l*_1_ (%)	5.8 (3)		6.6 (2)		3.7 (2)	

**Fig. 8:**
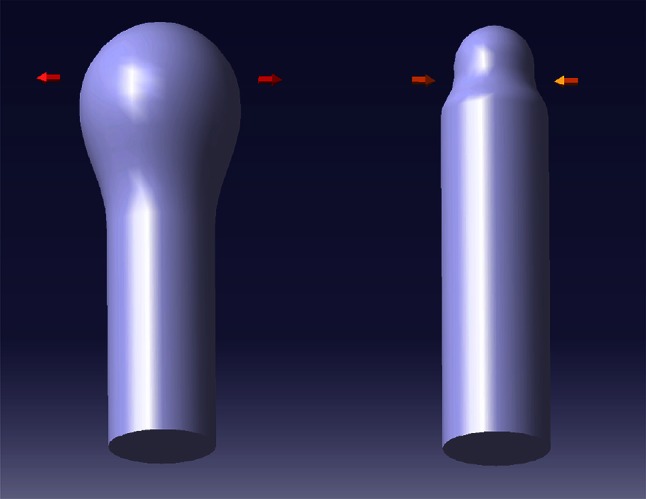
Schematic diagram of a basic vibration mode of the apical part of a pollen tube (exaggerated). Easy to imagine, in-phase, periodical mode allowing penetration through the tissue (thin or thick tip in each growth cycle)

**Fig. 9:**
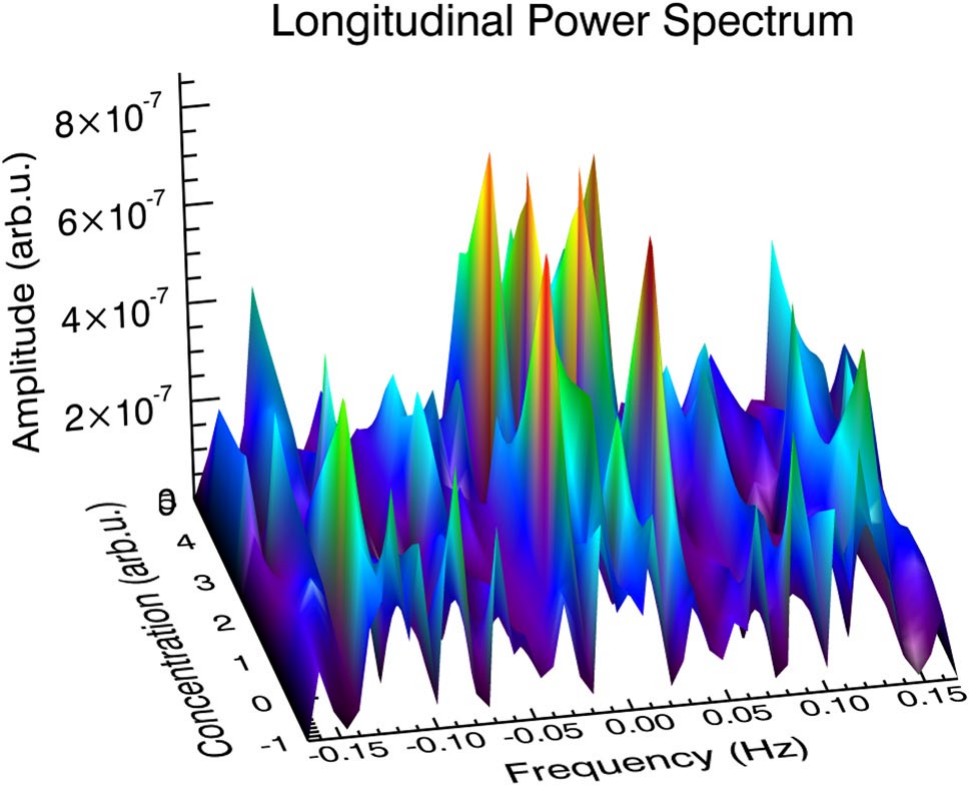
Longitudinal power spectrum of *Nicotiana tabacum* pollen tube: −1 corresponds to the hypotonic case, 0—isotonic case and 2.5, 3 and 5 correspond to 25, 30 and 50 mM NaCl for the hypertonic case, respectively. Low-lying peaks of high intensity are clearly visible. Interpolated by DAVE (Azuah et al. 2009)

**Fig. 10:**
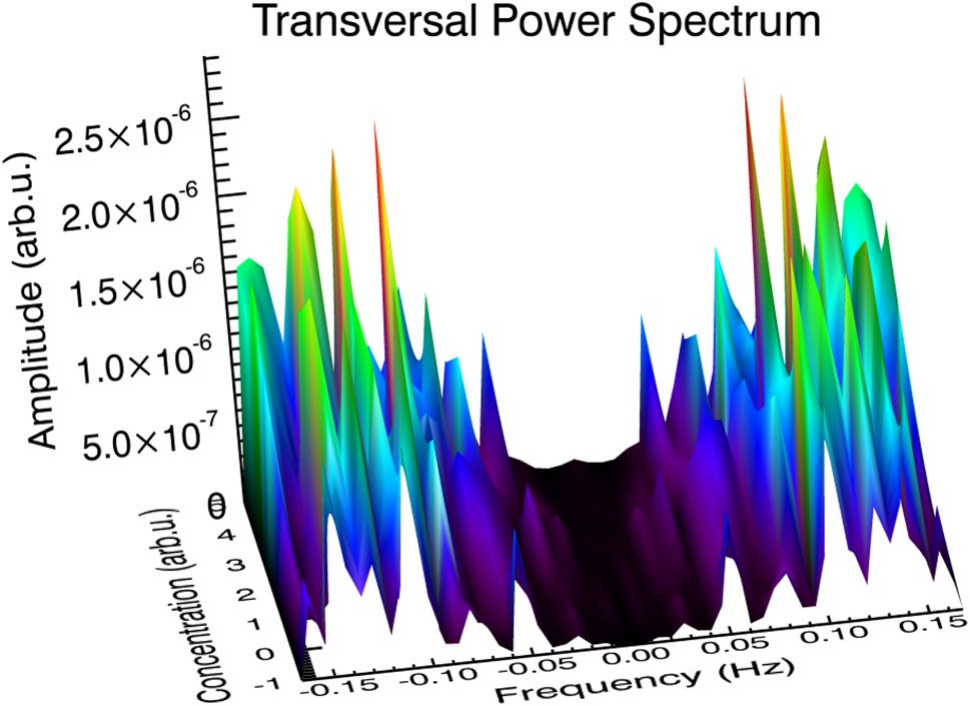
Transversal power spectrum of *Nicotiana tabacum* pollen tube: −1 corresponds to the hypotonic case, 0—isotonic case and 2.5, 3 and 5 correspond to 25, 30 and 50 mM NaCl for the hypertonic case, respectively. A gap at low-lying frequencies is clearly visible. Interpolated by DAVE (ibid.)

Figures 3, 4 and 5 show the energy density functions (obtained via FFT) generated from the signals presented in Fig. 2, related to the amount of energy dissipated at each frequency. Closer examination reveals relationship between the longitudinal and transversal spectra: In all transversal spectra a wide gap opens up, while in the longitudinal spectra some low-lying peaks exist (see also Figs. 9, 10), in the region marked as Ω_0_. Comparison with other authors (Zonia et al. 2006) show some similarities in the spectrum (existence of a peak centred about 0.15 Hz), but also discrepancies. In all figures (ibid., Figs. 7b, c, 8b, c, h, i, k, l, 9h, i, k, l, 10b, c) an intense, broad peak at zero is indicated and discussed later in the terms of 1/*f* noise. We must note that such procedure must be taken with caution as the noise should be based on the detrended spectra and analysed over wider frequency range. Direct comparison of peak subtle structure in both axial and transverse spectra leads to clear identification of significant peaks in all osmotic environments (Figs. 3, 4, 5, compare upper and lower charts for frequencies). Further procedure (linear regression) allows precise indication of fundamental oscillation frequencies in all cases of interest and low-lying higher harmonics (Figs. 3, 4, 5, the insets), which are determined with *R* ^2^ values >0.99.

Starting from the identified peaks with non-zero amplitudes, using the inverse Fourier transform (see Figs. 3, 4, 5; Table 3), we were able to retrieve from noise hidden time-domain periodicity. As the relative phase was lost in the analysis, we assumed no phase shift between the harmonics. These qualitative results, are clear enough to interpret, and are presented in Fig. 6. The change of oscillation periods is clearly visible either in rows [longitudinal (*L*) to transversal (*T*) transition], or columns (hypertonic–isotonic–hypotonic transition). These tendencies are visualized for the expanding or shrinking periods in all cases.

For further reconstructions, we limited ourselves to several basic harmonics, to visualize a general form-factor (a basic signal) of oscillations. As it is shown (Fig. 6, the insets), the form-factor is a triangle-shaped one (jigsaw). In the first approximation, the slope of both sides of these saw-shaped triangles may be used for discrimination of cell wall relaxation rates connected with varying magnitudes of internal pressure. (Special attention is required with peaks close to zero frequency in the longitudinal spectra, which may be connected with the exocytosis/elongation rates). In other words, the slope of the triangle may serve as a measure of the influence of turgor pressure-induced longitudinal and transversal oscillations onto wall building/relaxation processes and vice versa. In fact, both processes cannot be treated separately, which is encapsulated in the second non-trivial component (plastic-fluctuating) of the spectral density of the Ortega equation. We can say, that pressure relations and cell volume oscillations due to wall building/relaxation processes (in pollen tube apical region) are integral components of the biomechanics of *N. tabacum* pollen tube growth. They are clearly competing for the mechanical contribution to the total energy (*pV*) but with different shapes of spectral density. The wall building wants its energy centred at *f* = 0 (component 1), while the oscillatory part requires *f* > 0 (component 3). The tug-of-war is done through the plastic-fluctuating component 2.

Hence, we may expect, that the changes in turgor pressure may also influence pollen tube oscillations. In fact, it has already been observed (Taiz 1984) when the reduction in turgor pressure of only 0.02 MPa resulted in the immediate cessation of growth in the living cells. This led to the recognition (theory of loss of stability, Wei and Lintilhac 2007) that with an increase of turgor pressure the resulting stresses of the wall will gradually increase to a certain critical value, at which time loss of stability must occur, leading to stress relaxation of the wall, and consequent growth. We must note that close to the critical point there must be a boost for fluctuations which would promote the oscillations. Combining these elements together we may generally conclude that the polar growth of highly elongated cell of pollen tube requires self-consistent organization of many cellular features and functions.

To investigate closer the order of events of the pollen tube apex the cross-correlation analysis was performed between the *T* and *L* components. In the search for the sequence of events it turned out (Fig. 7) that in the hypertonic spectrum the transverse component is behind (negative time shift) and in hypotonic before (positive time shift) the longitudinal part. The isotonic case, although not clearly seen, must lie between those two cases with no shift between *L* and *T* modes.

A suitable explanation of biological mechanism behind positive or negative time delays between *L* and *T* modes (Fig. 7) can be given on purely mechanical foundations, assuming different and non-linear responses of the cell tip and basal part to the pressure. It has been shown earlier by Zerzour et al. (2009) (see Fig. 1 therein, and Fig. 3a for normally growing pollen tube) that under hypertonic conditions growth seizes except small swelling of apical region, meaning that the tip effectively expands faster than the distal part. In the opposite case of a higher internal pressure, the tip would reach its maximum plasticity (a plateau), while the response of distal part would continue to increase (see Fig. 1e, f ibid.).

We have to stress that despite being on the resolution limit of our microscope the high correlation between *T* and *L* modes cannot be generated by the random fluctuations of the image. From this data, a new picture emerges: in one cycle of the longitudinal wave measured in the apex, a transversal cycle takes place (see Fig. 8 for illustration). This situation, which is only observed in isotonic conditions may be termed as normal oscillation mode of the apical part of a pollen tube, see also Fig. 1A′, B′ in Pietruszka et al. 2012. This behaviour can be identified in Rounds et al. 2010, Fig. 3a, Fayant et al. 2010, Figs. 1, 4, 6 and Krichevsky et al. 2007, Fig. 2.

The common feature of all tip-growing cells is their ability to invade a biological matrix (Geitmann 2010; Sanati Nezhad and Geitmann 2013). As noticed, such invasion requires overcoming the mechanical impedance of the invaded matrix. And further: “To be a successful invader, any waste of energy needs to be avoided” (Geitmann 2010). By restricting cell expansion to the tip of the cell the potential energy loss is minimized (Fig. 8), where (phase 1) elongation takes place with the more pointed (shrinking) tip, which is followed by (phase 2) extension/swelling in the tissue without advancing in the axial direction. This mode of elongation being energetically and environmentally advantageous in vivo, is in agreement with our previous energetic considerations, also allowing for optimal efficiency of pollen tube penetration of stylar tissues. This must result in the appearance of the transverse oscillations and again advocates for their inclusion in the analysis. Eventually, we should note that the subtle spectrum—individual frequencies indicated by the pronounced peaks in the power spectrum (see Figs. 9, 10)—may be ultimately connected with physiological processes taking place in the growing pollen tube (e.g. calcium, chloride, etc. fluxes).

Rapidly growing pollen tubes show distinct similarities to the physics of phase transitions in that the system tends to become “quasi equilibrated” in extension under isotonic conditions, as opposed to both the hypo- and hyper-tonic conditions, where it expends more energy for less growth. It may be concluded that the freely growing cell is at the ‘critical point’, where the growth is the fastest. The presence of the self-organization (the correlation length must be at least of the order of the cell) of the system might be the reason behind the close to 1/f noise spectrum observed earlier (e.g. in Zonia et al. 2006), although we do not clearly see it in our data. This means that under normal growth conditions the system will tend to equilibrium (isotonic case) from hypertonic or hypotonic cases by equating osmotic potentials. Yet, this can be achieved by water and solute fluxes in or out of the growing cell. Furthermore, the fact that the calculated total energy is the lowest and that the linear velocity is the fastest in the isotonic case may indicate perfect adaptation of elongating (tobacco) pollen tube to the natural growth conditions.

## Conclusions

The fastest growth of the pollen tube apex under isotonic conditions cannot be adequately interpreted using only the longitudinal mode of oscillation. The importance of the transverse oscillatory component was confirmed by the analysis of its energy, which was 10 times higher than the longitudinal one. Under the isotonic condition, the transversal wave had the smallest amplitude. The least action principle was suggested as a possible candidate for the underlying law of nature behind such behaviour.

This work shows that the familiar Ortega equation can be re-interpreted using the methods of Fourier analysis to reveal the underlying energy/power relationship governing the rapid growth of single tip-growing cells such a pollen tubes. The elastic part was shown to be capable of promoting an oscillatory solution with a non-zero frequency even from a purely random fluctuations. At the same time, it is found to compete for spectral density with the plastic “steady growth *f* = 0″ component responsible for the elongation. Interestingly, both types of pollen extension, oscillatory and steady, are observed, and the transition between both cases should also be found. Finally, we have to stress that the analysis of the energy spectrum of the Ortega equation revealed presence from all contributions: the turgor pressure, fluctuations and cell wall elastic and plastic components with a simple but non-trivial coupling terms.

## Electronic supplementary material

Below is the link to the electronic supplementary material.

Supplementary material 1 (DOC 125 kb)
