# Supplementary material for: Power spectrum, growth velocities and cross-correlations of longitudinal and transverse oscillations of individual Nicotiana tabacum pollen tube
Source: Planta. 2014 May 11;240(2):263–76. doi: 10.1007/s00425-014-2083-5 (PMC4107278; doi:10.1007/s00425-014-2083-5)
Supplement: Supplementary file 1 — Supplementary material 1 (DOC 125 kb) [file 425_2014_2083_MOESM1_ESM.doc]

Supplementary Information
General: The experimental procedure was to analyze the response (in real time) of single pollen tubes to changes in the extracellular osmotic potential. To do these experiments, we had to develop a technique to immobilize the cells so they cannot move during the experiment. We developed (AH-S) method to grow the cells in an agarose gel medium directly on microscope slides. We had to measure and record growth in populations of pollen tubes using different concentrations of agarose. The results showed that the concentration of 0.3% agarose gave the best growth results but still kept the cells immobilized. Next we had to test how long it would take for the osmotic treatments to penetrate through the agarose medium. It was found that the osmotic treatments diffused through the agarose medium within 12-15 seconds. That was rapid enough for the live cell studies.

The Zeiss Axiophot upright microscope, connected to an UltraPix CCD (iso- and hypo-tonic cases) or Nikon DXM1200 (hypertonic case) cameras, were used for observing the cells. The microscope and camera were run by computer software (UltraView or NSI Elements). The experimental method involves recording fast time-series images of the live pollen tubes before and after osmotic treatment. Microscope images were collected at a rate of 1.8 images per second for UltraPix and twice as fast for NSI. Later on, the DXM1200 recordings were analysed only at every second picture, bringing it down to Ultrapix rate. The cells were recorded for 5 minutes before treatment, then a specific osmotic treatment was added and refocused on the same cell (this took from 30 seconds to 1.5 minutes), and then the pollen tube growth response was recorded for 10 minutes. At the end of each treatment, there were on average 1600 usable images for UltraPix case. These images were saved and transferred to a different computer for further analysis. The growth rate of the cells using the open-source morphometry software ImageJ, from the images at 3 second intervals. Growth rate was measured by choosing a fixed point on the cell, and then measuring the distance to the apex. These measurements gave a list of lengths (micrometer) that were imported into Excel. The exact time intervals were transferred from UltraView, which were imported into the same worksheet in Excel. From the length and time measurements, the growth rate was calculated using the formula (x2-x1)/(t2-t1), with x - length and t - time.
Measurement details:

To perform measurements the following equipment was used:
·	Microscope: Axiophot – ZEISS upright microscope Germany (Conventional upright microscope used for high resolution colour imaging, also in combination with fluorescence).
·	Objective: Achroplan 63x/0.95 W Ph3 ∞/0 (water immersion)

In the hypo- and iso-tonic case, we utilized: UltraPix CCD camera, Ultrapix 1600: 1536x1024 pixels

In the hyper-tonic case, we utilized:
·	Camera: Nikon digital camera DXM 1200. High resolution, 12.6 megapixel digital photomicrographic camera captures the sharpest images flawlessly.

The Digital Eclipse DXM1200C is an ultra high-quality digital camera for advanced imaging. It features a new Peltier cooling mechanism to significantly reduce heat induced noise, improved image resolution, and new software to enhance camera control, digital viewing, colour rendition, and capture of images in a variety of applications. This model continues the DXM tradition of capturing images with user selectable high pixel count image files for documentation and high resolution image printing capabilities. The DXM1200C can capture image files with up to 12.6 million pixels, using Nikon's exclusive pixel stepping technology, making it an ideal choice for applications requiring the highest spatial resolution possible. http://www.nikoninstruments.com/Products/Cameras/Digital-Cameras/DXM-1200C DXM 1200 is used in for situations where extreme resolution photography is required.  http://www.pdis.org/equipment/ScopeCameras.aspx

Objective: N-Achroplan 
Magnification: 63x
Numerical Aperture: 0.95 (in air), 1.26 (water)
Coverglass Thickness [mm]: 0
Immersion: Water immersion

We worked with the Zeiss Axiophot upright microscope, connected through a Nikon, DXM 1200 CCD camera, that we used for observing the cells.  We used long-distance microscope lens (Achroplan, Zeiss, Germany). The lens of magnification factor 63x was utilized with a numerical aperture (NA) of 0.95.
https://www.micro-shop.zeiss.com/?l=en&p=us&f=o&a=v&m=a&id=420984-9900-000
Due to water immersion the effective NA was equal to 1.26. 

·	Program: NSI – Elements AR3.0 (for camera manipulation and recording) 

For further analysis 3 s interval was chosen 3300 images (twice frequency of UltraPix) were taken in each series in .nd2 Nikon format (not compressed), from which half was skipped was analysed with imageJ program with Bio-Formats.

Bio-Formats is a standalone Java library for reading and writing life sciences image file formats. It is capable of parsing both pixels and metadata for a large number of formats, as well as writing to several formats. See the table for a complete list http://loci.wisc.edu/software/bio-formats

In program NSI – Elements AR3.0 the following parameters were tuned: image sharpness and brightness: 250 ms, grain: 140. 
Program format for capture: 640x512 focus. 
Duration: no delay.
Loops: ok 3300 ± 1 (images). 
Time: 900 ± 0.01s
Nikon DXM 1200 camera was selected > mono camera (emulation) – shades of gray. 


Measurements resolution adjustment:

Pollen tube growth measurements precision

·	Introductory scaling (aspect ratio calculation in the transition from UltraView to imageJ)

Lp	Witdh	UltraView [ìm]	imageJ [px]	Aspect ratio
[px/ìm]	[ìm/px]	
1.	horizontally	9.97 ± 0.01	48.042 ± 0.001	4.819	0.208	
2.	horizontally	9.76 ± 0.01	48.781 ± 0.001	4.998	0.200	
3.	horizontally	9.54 ± 0.01	45.051 ± 0.001	4.770	0.210	
4.	vertically	61.15 ± 0.01	280.249 ± 0.001	4.583	0.218	
5.	vertically	60.90 ± 0.01	277.302 ± 0.001	4.553	0.219	
6.	vertically	61.16 ± 0.01	278.492 ± 0.001	4.553	0.220	

 aspect ratio (mean) = 4.713 [px/ìm] and 0.213 [ìm/px]
*px – pixels 

·	Exact scaling

No.	Aspect ratio [ìm/px]	
1	0.21	
2	0.23	
3	0.21	
4	0.23	
5	0.23	
6	0.23	
7	0.23	
8	0.23	
9	0.23	
10	0.23	

Ratio = 0.226 ≈ 0.23 [ìm/px]

Length [px] x 0.23 = length [ìm]

By using Nikon, DXM 1200 CCD camera and  NSI – Elements AR3.0 program the final 10-times  scaling was performed which finally gave the ratio = 0.23 [ìm/px]. The measurements of growth/growth rate were performed in imageJ program.

Data from imageJ were transferred to Excel, where growth rate was calculated in longitudinal and transversal directions from the following formula GR= (l1-l0)/(t1-t0), where:  l - length [ìm], t -  time [s]. For longitudinal GR the bias (mean velocity) was subtracted before performing FFT.


Oscillation frequency calculation

Fourier transformation [FFT] was used as described symbolically beneath

t	GR	
…	...	
…	…	

 FFT ↓

f	A	
…	...	
…	…	

to transfer from time t and GR to frequency f and amplitude A variables, which was performed in Origin 6.0 program by using Hann window.
